# Supplementary material for: Confirmatory factor analysis of the Tinnitus Impact Questionnaire using data from patients seeking help for tinnitus alone or tinnitus combined with hyperacusis
Source: PLoS One. 2024 May 8;19(5):e0302837. doi: 10.1371/journal.pone.0302837 (PMC11078403; doi:10.1371/journal.pone.0302837)
Supplement: Attachment — Submitted filename: Response letter - PloS.docx [file pone.0302837.s001.docx]

Dear Dr. Ravi,

We would like to express our gratitude for the opportunity to submit our revised manuscript titled " *Confirmatory factor analysis of the tinnitus impact questionnaire using data from patients seeking help for tinnitus alone or tinnitus combined with hyperacusis*" to PLOS ONE for consideration. We appreciate the constructive feedback provided by the reviewers and the editor, which we have considered in revising our manuscript.

In response to the reviewers' comments, we have made revisions to address the concerns raised, and these are highlighted in yellow in the text. Specifically:

1. Reviewer 1:

We thank reviewer #1 for their time to review the manuscript and positive feedback.

2. Reviewer 2:

Comment 1) Title - Tinnitus Impact Questionnaire should be capitalised.

Response 1): We have amended the title as requested.

Comment 2) Text - capitalise Visual Analogue Scale; not necessary to capitalise "Autistic Spectrum Disorders".

Response 2) The term "Visual Analogue Scale" has been capitalised and all instances of "Autistic Spectrum Disorders" have been converted to lower.

Comment 3) p. 7 and p. 15); p.9 "... on the number of days it occurred: ..." - specify that the number of days is over a 2 week period;

Response 3) Changed as suggested.

Comment 4) p. 12 subtitle "Discriminant and convergent and validity" needs correction;

Response 4) The text has been corrected.

Comment 5) give a reference for the 'lavaan' package and briefly describe it in the text (an extra phrase will suffice);

Response 5) A reference has been added, and a brief description has been added.

Comment 6) p. 23 delete comma in "Although, it has long been ..."

Response 6) Comma has been removed.

Comment 7) References - 1. No need for "The"; 5. should be "HNO"; 7. Should be "Tinnitus Functional Index"; 11. delete "/the British Psychological Society"; 13. journal title change to "Journal of Neuroscience"22. typo ". . "; 25 no capital needed for "People"; 32. typo "; 1997", give report number; 41. "1, editor" and ".;" typos; 44. capitalise "Tinnitus Handicap Inventory"; 45. full stop needed after title; 47. typo "5th ed ed."; location of publisher needed; 61. "The" not required in title; 64. should be " .. Tinnitus Functional Index ..."; 67. volume and page numbers absent; 73. capitalise test name. In general, check all references and be consistent in how titles are presented (some are in full, others are abbreviated - use the PLOS ONE recommended style).

Response 7) We have downloaded the PLOS ONE EndNote citation style and updated the reference list accordingly.

Thank you for your time and consideration.

Sincerely,

Mercede Erfanian (PhD)
